# Recruitment and Retention Techniques for Developing Faith-Based Research Partnerships, New York City, 2009–2012

**DOI:** 10.5888/pcd10.120142

**Published:** 2013-03-07

**Authors:** Jessica M. Hippolyte, Erica G. Phillips-Caesar, Ginger J. Winston, Mary E. Charlson, Janey C. Peterson

**Affiliations:** Author Affiliations: Erica G. Phillips-Caesar, Ginger J. Winston, Mary E. Charlson, Janey C. Peterson, Weill Cornell Medical College, New York, New York.

## Abstract

**Background:**

Faith-based organizations are recognized as an influential venue for behavioral health interventions. However, less is known about efficient approaches for identifying and recruiting these organizations and about the processes that enable successful partnership.

**Community Context:**

In 2007, 66% of Latinos and 70% of blacks in New York City reported being overweight or obese. Project SCALE (Small Changes and Lasting Effects) is a 5-year randomized behavioral weight loss intervention trial aimed to help black and Latino adults lose weight by making small changes in eating behaviors and daily leisure physical activity. The study partnered with faith-based organizations.

**Methods:**

Faith-based organizations were identified primarily through direct referrals. Recruitment consisted of screening faith-based organizations, establishing a memorandum of understanding, and intervention modification. Partnership maintenance occurred primarily via progress meetings.

**Outcomes:**

We identified processes that supported and impeded study recruitment and retention. Obtaining leadership support and using group orientation sessions were successful recruitment and retention processes. A balance must be found between leadership, advocacy, and causing members to feel pressured to participate in the study.

**Interpretation:**

Behavioral health interventions implemented in faith-based organizations can reduce health disparities. However, researchers must determine whether faith-based organizations have the capacity to partner in intensive interventions. Focusing on the establishment of strong partnerships at the onset will help ensure that mutual objectives are achieved and sustained long-term.

## Background

The prevalence of overweight and obesity continues to be a public health problem in the United States (1,2). Although the rates of overweight and obesity have increased in all racial and ethnic groups, black and Latino populations have a disproportionately higher prevalence of obesity and obesity-related diseases such as diabetes and cardiovascular disease, making this a substantial public health concern (3).

Faith-based organizations (FBOs) have been recognized as an influential venue for developing and implementing disease prevention and health promotion efforts (4). Interventions targeting obesity and cancer prevention in predominantly African American churches have been successful (5,6). Fewer nationwide efforts have been made in Latino communities, even though 90% of Hispanics are members of a church or faith-based group (7). To successfully increase efforts in using these venues, researchers need more detailed understanding of efficient approaches for identifying, recruiting and developing a process for partnering with these organizations.

## Community Context

In 2007, 66% of Latinos and 70% of blacks in New York City (NYC) reported being overweight or obese. Of these adults, 74% reported not engaging in recommended physical activity levels. NYC neighborhoods with the highest rates of obesity are also the poorest; approximately one-third of the population lives below the federal poverty level (8). Furthermore, black New Yorkers were 3 times as likely to die of diabetes and Latinos were twice as likely to be diagnosed with diabetes as whites (9). Thus, new strategies are needed to enable sustainable weight loss in these vulnerable populations.

Project SCALE (Small Changes and Lasting Effects) is an ongoing study funded by the National Heart, Lung, and Blood Institute that is refining and testing a small-change approach to obesity intervention, targeting overweight or obese black or Latino adults who reside in Harlem or the South Bronx. Participants select a small-change eating strategy, such as using a 10-inch plate or making half their main meal vegetables, along with a self-selected physical activity goal. This approach is coupled with a positive affect and self-affirmation intervention (10). Participants are enrolled for 1 year and are followed by a community health worker (CHW) who assists them in achieving their goals. This study was approved by Weill Cornell Medical College and Lincoln Medical and Mental Health Center institutional review boards. This article is based on the study’s pilot and early phases of the randomized clinical trial and focuses on lessons learned in the recruitment and retention of community-based participants through FBOs.

## Methods

### Engagement of faith-based organizations

Project SCALE engaged a convenience sample of 5 FBOs in the South Bronx and Harlem communities through direct referrals from community-based partners (Figure) and online research using Google maps. The work was conducted during a prestudy period and 3 subsequent years (Table 1). The Google search yielded approximately 400 FBOs in Harlem and approximately 240 in the South Bronx. Initial telephone contact with those FBOs led to about a 0.06% yield on returned telephone calls; this method was discontinued. We established 3 processes for engaging FBOs: 1) FBO screening to assess demographics, infrastructure, and physical capacities; 2) establishing a memorandum of understanding; and 3) modifying the intervention.

**Figure:**
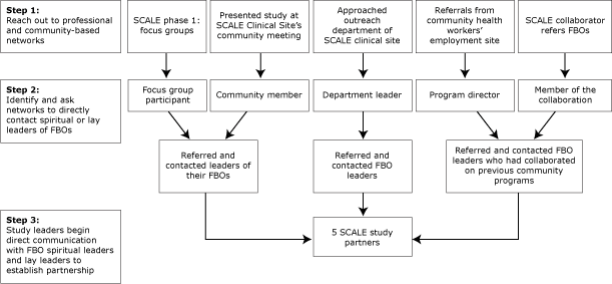
Faith-based organization (FBO) identification process. Abbreviation: SCALE, Small Changes and Lasting Effects.

**Table 1:** Projected Timeline to Establish Faith-Based Research Partnership, Project SCALE, New York City, 2009–2012

Step	Year and Quarter													
	2009				2010				2011				2012	
	1	2	3	4	1	2	3	4	1	2	3	4	1	2
**Phase 1: identification of FBOs**														
Referrals from community partners			X											
Direct contact			X											
**Phase 2: recruitment of FBOs**														
FBO screening				X										
Establishing memorandum of understanding				X										
Intervention modification				X										
**Study duration (enrollment to close-out)**					X	X	X	X	X	X	X	X		
**Phase 3: development and maintenance of partnership**														
Progress meetings					X	X	X	X	X	X	X	X		
Offer FBO members a position as a community health worker				X										
Plan of action (See Box for details)					X	X	X	X	X	X	X	X		
Assessment of approach													X	
Dissemination of results														X

Abbreviations: SCALE, Small Changes and Lasting Effects; FBOs, faith-based organizations.

### FBO screening

We sought partnerships with FBOs who self-identified their membership as being primarily black or Latino adults, had varying membership size, and had internal and external FBO activities. The latter 2 components were important to the potential reach of the project outside of the FBO. Most social networks are demographically homogeneous, meaning people generally interact with those like themselves (11). FBOs that dominate their members’ time and discourage contact with secular society can restrict interactions among demographically diverse people, particularly between people within the FBO and those outside of the FBO (12). FBOs with external activities have the opportunity to create more heterogeneous networks and may encourage members to participate in extra FBO activities (13). Our partnering FBOs had varying demographic characteristics (Table 2).

**Table 2:** Faith-Based Organization (FBO) Demographics, Project SCALE, New York City, 2009–2012

Characteristic	First Baptist Church	St Luke’s Catholic Church	Metropolitan Community United Methodist Church	Abyssinian Baptist Church	Iglesia Congregación Cristiana del Bronx
Neighborhood	Harlem	Bronx	Harlem	Harlem	Bronx
Denomination	Baptist	Roman Catholic	Methodist	Baptist	Nondenominational
Primary race/ethnicity	Black	Latino	Black	Black	Latino
Membership size	>300	>300	<300	>300	<300
Age range	Youth to elderly	Youth to elderly	50% of members aged ≥35 years	Youth to elderly	50% of members aged ≥35 years
Activities	Choir practice, Bible study	Rosary groups, food pantry, school on premises (kindergarten–8th grade)	Bible study, feeding ministry, Monthly Health Moment, annual health fair	Bible study, 70 ministries (monthly meetings), Health Program Kickoff Events, Health Fair, Go Red Event	Bible study, Bible school, intercession prayer service and worship celebration, evangelistic walkout, youth meetings, health fair, evangelistic day
Health ministry present	No	No	Yes	Yes	Yes
No. of FBOs within a half-mile radius	>20	15–20	>20	>20	10–15

Abbreviation: SCALE, Small Changes and Lasting Effects.

We also assessed the FBO’s infrastructure (Box). We identified 3 structural components that were important to establishing the partnership and enhancing study recruitment and retention. The first was the presence of a lay leader, which was the first person of contact at all 5 participating sites. Study personnel contacted and organized a meeting with each lay leader to introduce the study, answer questions, and provide lay leaders with enough time to discuss the feasibility of implementing the intervention with the organizations’ spiritual leaders. The second component was the presence of regularly programmed activities within the FBO. During preliminary discussions, ministry leaders and study personnel agreed that study activities should not occur at times of scheduled worship services. Thus, the best time to recruit participants was during weekly or monthly programming such as ministry meetings, rosary groups, Bible studies, choir practices, major church events (eg, health fairs) and community events in which the FBO is a key participant (eg, Annual Central Harlem Health Revival). During these activities, CHWs were present before, during, and after the activity to provide study information and conduct enrollment and follow-up surveys; at times, they were active participants. CHWs did not interrupt the logistics of these activities, but their presence increased their ability to build a relationship with potential participants and those already enrolled. The third component was evaluating the organization’s physical capacity to determine whether space was available to hold meetings, conduct surveys and other study-related events, and to uphold research principles such as confidentiality and privacy.

**Box. Methods of Faith-Based Organization (FBO) Identification and Partnership Maintenance, Project SCALE (Small Changes and Lasting Effects), New York City, 2009–2012**

**Engagement of FBO**

1. FBO screening
  - Demographics
    - Race and ethnicity
    - Membership size
  - Infrastructure
    - Presence of lay leaders
    - Programmed activities
    - Membership’s participation
  - Physical capacity
    - Enrollment space
    - Privacy
2. Establishing a memorandum of understanding
  - Expectations from FBOs
  - Expectations from Project SCALE
3. Intervention modification
  - Faith-based versus faith-placed
    - Inclusion of Biblical scriptures in study guides
  - Recruitment and retention plan
    - Individual recruitment
    - Group orientation sessions
    - Pastoral advocacy
    - Study presentation to FBO members
    - Study personnel attend FBO activities
    - Service attendance by study personnel
      - Principal investigators
      - Coordinators
      - Community health workers
    - Health fairs
    - Study advertisement in weekly bulletin
    - Study-specific (with study logo) flyers, brochures, newsletters, water bottles, tote bags

**Development and Maintenance of FBO Partnership**

1. Progress meetings
  - Recruitment and retention update
  - Reassessment of approach
  - Modifying approach as necessary
  - Soliciting feedback from clergy, lay leader, and congregation
2. Offer FBO members positions as community health workers
  - FBO leaders announce and refer members
  - Study staff hire and train members
  - Community health workers recruit at respective FBO
3. Plan of action
  - Service attendance
    - Principal investigators
    - Coordinators
    - Community health workers
  - Provision of monetary funds
  - Study personnel attendance at FBO activities
  - Study personnel volunteer
    - Food ministry
    - Food pantry
  - Health presentations
  - Additional contributions
    - Health fair supplies and personnel

### Establishing a memorandum of understanding

Although we did not use a community-based participatory research approach in all facets of the study, we did incorporate several guiding principles (14). For example, we used memorandums of understanding (MOUs) to help formalize the roles, responsibilities, and activities of academic and community partners. The MOU also delineated who was accountable for specific tasks and activities as well as for the overall project (Appendix). The MOU was given to the leadership board for review, approval, and signature. This method did not meet any barriers and provided a solid framework for the partnership.

### Modifying the intervention

To ensure successful uptake and a sustainable intervention at each FBO, members of the FBO who attended planning meetings examined study materials, explored the methods of recruitment and ongoing support, and helped to further adapt the intervention to fit their memberships’ need. Suggestions that came from the group included incorporating scriptures into educational materials, which included descriptions of the risk factors associated with obesity, theory behind the small-change approach to weight reduction, and ways to increase physical activity. The Box lists other recommendations used in tailoring the intervention.

This approach incorporated some of the guiding principles of community-based participatory research. Intervention modification enables the FBO to significantly contribute to pre-implementation. Furthermore, this early planning decreases the likelihood of setbacks during implementation, because FBO leadership and study personnel have already discussed and agreed on most processes (eg, how to promote the study, most efficient days and times to recruit, best activities for CHWs to attend). This planning presents the study as a united effort between study personnel and FBO leadership, conveys to the FBO membership that the intervention is catered toward their needs, and can make the membership more receptive.

### Development and maintenance of FBO partnership

Spiritual leaders, lay leaders, and study staff met at least quarterly to discuss ideas and strategies to enhance recruitment and retention. During the introductory meetings, study staff discussed the opportunity to employ and train a member of the FBO as a CHW. Study staff circulated a written job description among congregates. Only 2 of the 5 FBOs put forth members with appropriate qualifications for the position. The interview process resulted in the successful hiring and training of at least 1 CHW from a partnering FBO. Additional methods were used to maintain a collaborative relationship with the FBO (Box).

Study maintenance is enhanced because the intervention had minimal costs. Study-related expenses (salary for trained FBO member; study materials such as guides, scale, tote bags, and water bottles) are covered through federal grants. With the exception of the guide, all other materials distributed to FBO members are gestures of appreciation for study participation and are not vital for the study’s continuation or program implementation in other FBO sites. Fundraising activities in FBO sites would cover the cost of printing study guides. In addition, the promotion and adoption of the study’s eating and physical activity strategies can continue without cost to the FBO or its members. The study was designed so that all eating and physical activity strategies are inclusive and can be adapted to anyone regardless of socioeconomic status. For instance, because 1 strategy includes increasing vegetable intake, we emphasize using fresh, frozen, or canned vegetables to prevent exclusion. Furthermore, we recommend increases in daily lifestyle physical activity such as walking and climbing stairs because they are low cost.

### Assessment of approach

On study completion, quantitative data such as attrition rates will be used to assess our recruitment and retention approaches. Qualitative data will also be collected at participants’ close-out interviews to understand their perception of recruitment and retention methods such as group orientation sessions. Focus groups will be held with all lay leaders and clergy. We will disseminate results to the FBOs’ leaders, members, and community partners when the assessment is completed.

## Outcomes

### Pastoral support

Although lay leaders provide an entry point into the FBO, the intervention’s implementation is contingent on full support from the pastoral leaders. In addition to support, the spiritual leaders should directly advocate the study to their congregation. In 1 FBO where the spiritual leader offered full support, we saw higher rates of retention (92%) during the 12-week pilot than in an FBO where the pastoral leader approved of the study but was not outwardly supportive of the program (62%).

The actual participation of FBO leaders in the intervention exemplifies leading by example. For instance, the spiritual leader of 1 FBO enrolled in and completed the 12-week pilot study. This allowed the spiritual leader to provide an internal perspective, which reduced stigma associated with study participation, decreased fear and misconceptions, and motivated study participation among previously apprehensive members. However, we want to prevent burdening the FBO’s leaders with the expectation that they must participate, which may adversely influence the receipt and adoption of the intervention.

### Acknowledgment of precontemplation

Although pastoral support is instrumental in motivating members to take part in research, not all members will be moveable from a precontemplation phase. For instance, the strategy of 1 smaller FBO (<300 members) was for the pastoral leader to advocate for study participation during the worship service and ask members of the congregation to complete sign-up sheets immediately after the study announcement and before the continuation of service. CHWs received a compiled list from the leader indicating that 41 members expressed interest in the study; however, when directly contacted, only 13 members were interested. In this instance, the pastoral leader’s enthusiasm sent an unspoken expectation, which led us to contact people who were not prepared to make behavioral changes. In contrast, the methods used in a larger FBO (>300 members) was that the spiritual leader presented the importance of the study during worship service and advised those interested to seek out the CHW and study staff after service. The latter resulted in 90 people completing the study informational request, approximately 75% of whom completed the enrollment process. This approach enabled people to make a more informed decision about enrolling in the study.

### Group orientation sessions

During the last 20 years, retention rates for weight-loss trials have not improved substantially. Thus, we applied a motivational interviewing technique to diffuse ambivalence during interactive group-based orientation sessions in 2 of the partnership sites. We used the model developed by Goldberg and Kiernan (15) for the orientation sessions to address the following areas: 1) disparities of obesity and its health-related complications in the study population, 2) ambivalence about making eating and exercise behavior changes, 3) ambivalence about joining a randomized controlled trial, 4) goal setting for unrealistic weight-loss expectations, 5) the importance of a scientific control condition, 6) the concept of random assignment in clinical trials, and 7) the effect of dropouts from clinical trials. Participants were then divided into small groups and asked to generate 2 pros and 2 cons of being assigned to a control condition and an active condition. Participants shared their pros and cons with the larger group, while the investigator asked open-ended questions, engaged in reflective listening, and avoided taking a “pro-change” position. Of those who attended the orientation, 60% (site 1) and 75% (site 2) completed the enrollment process. At the end of the trial we will compare FBOs where a group orientation session took place versus those where it was not conducted to assess the effect on retention rates.

## Interpretation

The aim of the SCALE intervention is to achieve weight loss by encouraging small behavioral changes among blacks and Latinos. One barrier to engaging this population is the mistrust of research studies among racial/ethnic minority populations. Consequently, we sought partnerships with FBOs to access this population in an environment where they felt safe, which would enable us to increase recruitment and retention rates in a population underrepresented in the literature.

Although partnering with FBOs is a logical and feasible way to recruit and retain racial/ethnic minority populations, we learned several best practices for partnering with FBOs to achieve mutual study objectives. We compiled a checklist of important components and processes that may help those looking to engage in similar work. First, use networks when identifying FBOs because it provides an entry point; without a direct referral, the likelihood of establishing a partnership is low. Once FBOs are identified, they must be screened to determine whether the demographics (eg, race, ethnicity, age) of the FBO’s membership align with requirements for the intervention. Consider the infrastructure and assess whether there is a committed and supportive clergy and lay leader, regularly scheduled activities with steady attendance rates at which to interact with members, and enough physical space to accommodate study-related activities. The presence of a health ministry or group that already promotes health activities will increase the congregation’s receptivity to the study.

When it is established that the FBO has the foundation necessary for successful implementation, study staff and FBO leaders should outline and agree on expectations by way of an MOU. Furthermore, consider partnering with an FBO where the leaders are willing to engage in the modification of the intervention and remain active by participating in meetings to assess whether goals and objectives are being met. Our experiences illustrate that successful partnership and implementation is enhanced when health professionals approach a faith-based partnership with these components and processes in mind; a lack of preparation can impede study implementation, recruitment, and retention.

To maintain trust and transparency throughout all study phases, involve partners in modifying the intervention to suit their special needs, have FBO leaders participate in study planning meetings, and encourage study staff to volunteer in FBO events unrelated to the study. The ability to employ and train a member of the FBO to deliver the intervention not only promotes inclusion but also ensures that a voice from the FBO is always present. This member’s knowledge and training helps to ensure intervention longevity. Dissemination of results to FBOs and the community at large will ensure sustainability.

Our study had limitations. First, the lessons and methods presented here were discovered throughout study implementation, and thus we were unable to apply them to each FBO. Second, the collaborating FBOs did not represent a wide range of religious backgrounds and consequently the findings may not be applicable to all denominations.

Despite these limitations, the project illustrated that specific processes and characteristics can facilitate successful implementation of a behavioral health intervention. Community engagement is vital for successful uptake and sustainability of health interventions, and focusing on the approach and establishing strong partnerships from the beginning will help ensure that mutual objectives are met and sustained long-term.

## Appendix. Memorandum of Understanding.

This file is available for download as a Microsoft Word document.
