# Supplementary material for: Recruitment and Retention Techniques for Developing Faith-Based Research Partnerships, New York City, 2009–2012
Source: Prev Chronic Dis. 2013 Mar 7;10:E30. doi: 10.5888/pcd10.120142 (PMC3603629; doi:10.5888/pcd10.120142)
Supplement: Supplementary file 1 [file 12_0142Appendix.doc]

Appendix. Memorandum of Understanding Used in the Small Changes and Lasting Effects (SCALE) Trial, New York City.

Currently, The Small Changes and Lasting Effects (SCALE) Trial seeks commitments from key stakeholders and organizations serving the residents of the South Bronx and Harlem community to collaborate in this research project to serve as facilitators and dedicate time and commitment in attending meetings as necessary to support research participants.

The SCALE Trial is a collaboration between Weill Cornell Medical College, Cornell University, Lincoln Medical and Mental Health Center, Renaissance Health Care Network, and Northern Manhattan Perinatal Partnership. The SCALE trial is funded by the National Heart, Lung and Blood Institute of the National Institute of Health (NIH).

The purpose of the SCALE trial is to focus on promoting small changes in eating behavior and increasing physical activity among Black and Latino participants in Harlem and the South Bronx. The desired result from these activities is weight loss that will be sustained years after participation in the study. The goals of the SCALE trial are:

1) To explore and build a better understanding of how culturally different groups perceive making changes in eating and physical activity behavior.

2) To examine the utility of making small changes in eating behaviors and physical activity in Black and Latino participants in Harlem and the South Bronx. The goal is individual weight loss of at least 7% in one year.

3) To test if positive thinking can protect participants against the negative impact that stress and depression can have on making positive behavior changes.

4) To develop a means of generalizing this effort to other Black and Latino populations in other urban communities.

Expectations for Faith Based and Community Organizations involved in SCALE

• Obtain faith based and community organization leadership support

• Promote study and encourage congregation/community organization to participate

• Designate a representative from the organization to participate in study planning meetings (planning meetings occur quarterly and are held at the medical college on 68th street and York avenue)

• Develop tailored ideas for recruitment and retention of participants from your organization

• Assist in planning an event at your organization such as a health fair (paid for by study)

• Support community health worker assigned to the site and assist in keeping participants enrolled in the study for the full 12 months

Expectations for the SCALE Project Team,

• Hire, train and provide Community Health Workers assigned to the faith based organization

• Recruit participants

• Complete study related assessments like questionnaires, phone interviews, etc

• Provide and answer study related questions

• Convene and direct monthly support meeting

• Monitor the progress of all SCALE related activities

• Draft all project communications and reports

• Facilitate communication to the community base organizations

On behalf of myself/organization I will participate in the SCALE Project.


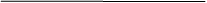

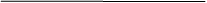


Signature & Date Signature & Date


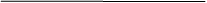

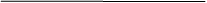


Printed Name Printed Name


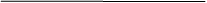

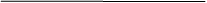


SCALE Representative Faith Based or Community Organization Representative

Weill Cornell Medical College

Center for Integrative Medicine

Division of Clinical Epidemiology &

Evaluative Sciences Research
